# Cognitive Impairments Related to COMT and Neuregulin 1 Phenotypes as Transdiagnostic Markers in Schizophrenia Spectrum Patients

**DOI:** 10.3390/jcm13216405

**Published:** 2024-10-25

**Authors:** Mariana Bondrescu, Liana Dehelean, Simona Sorina Farcas, Ion Papava, Vlad Nicoras, Carla Andreea Podaru, Madalina Sava, Elena Sabina Bilavu, Sandra Putnoky, Nicoleta Ioana Andreescu

**Affiliations:** 1 Department of Neurosciences-Psychiatry, “Victor Babes” University of Medicine and Pharmacy, Eftimie Murgu Square 2, 300041 Timisoara, Romania; mariana.bondrescu@umft.ro (M.B.); papava.ion@umft.ro (I.P.); 2 Timis County Emergency Clinical Hospital “Pius Brinzeu”, Liviu Rebreanu 156, 300723 Timisoara, Romania; vnicoras@gmail.com (V.N.); sava.madalina87@gmail.com (M.S.); sabinabilavu@yahoo.com (E.S.B.); sandra.putnoky@yahoo.com (S.P.); 3 Doctoral School, “Victor Babes” University of Medicine and Pharmacy, Eftimie Murgu Square 2, 300041 Timisoara, Romania; 4 Discipline of Medical Genetics, Department of Microscopic Morphology, Center of Genomic Medicine, “Victor Babes” University of Medicine and Pharmacy, Eftimie Murgu Square 2, 300041 Timisoara, Romania; farcas.simona@umft.ro (S.S.F.); andreescu.nicoleta@umft.ro (N.I.A.); 5 Independent Researcher, 300523 Timisoara, Romania; carla.podaru@gmail.com

**Keywords:** cognitive impairments, neuregulin 1, COMT, schizophrenia spectrum disorders

## Abstract

**Background:** Research on the interaction between antipsychotic treatment and cognitive dysfunction in schizophrenia spectrum disorders (SSDs) is extensive, yet the role of genetic polymorphisms in catechol-O-methyltransferase (COMT) and neuregulin 1 (NRG1) remains underexplored. **Methods**: This study evaluates the impact of COMT (rs4680) and NRG1 (rs3924999 and rs35753505) polymorphisms on cognitive functions in SSD patients. A cross-sectional study was conducted with fifty-four patients, assessed using the Positive and Negative Syndrome Scale (PANSS) and the CNS Vital Signs battery. **Results**: Significant cognitive function differences were observed across SSD diagnostic categories (*p* < 0.001). The NRG1 rs35753505 TT genotype was significantly associated with better verbal memory performance compared to the CC genotype (*p* = 0.03), while no significant differences were observed for other genotypes. The NRG1 rs3924999 AA genotype showed superior reasoning performance compared to AG and GG genotypes (*p* = 0.01), with AG and GG associated with lower scores (*p* = 0.01 and *p* = 0.02, respectively). Additionally, the COMT Val158Met genotype significantly influenced processing speed, with patients at the first episode of psychosis showing higher scores than chronic patients (*p* = 0.01). **Conclusions**: These findings suggest that NRG1 and COMT polymorphisms may influence cognitive domains in schizophrenia spectrum disorders, potentially informing personalized treatment and cognitive rehabilitation strategies.

## 1. Introduction

Schizophrenia spectrum disorders (SSDs) encompass a range of conditions, with schizophrenia being the most well-known. While clinical symptoms can differ both between and within these disorders, certain common features are frequently observed across the various forms. Among these, cognitive impairments have been extensively studied, particularly in relation to schizophrenia and first-episode psychosis, where they are regarded as promising markers [1]. Conversely, the impact of schizoaffective disorder on cognitive function has received comparatively little attention [2]. Affective psychosis is generally associated with a better prognosis, though the evidence for cognitive impairments in this context is less well established. Nonetheless, cognitive impairments do appear in premorbid states and tend to stabilize as the disease progresses into a chronic phase. These impairments are of significant importance, given their early onset and substantial influence on the prognosis of psychosis.

Brief psychotic disorder, schizophreniform disorder, schizophrenia, schizoaffective disorder, and delusional disorder are the main representative diseases from the schizophrenia spectrum according to DSM-5 [3]. Psychopathological aspects, cognitive deficits, and motor symptoms affect patients’ functionality, prognosis, and treatment response [4]. Although hallucinations and delusions interfere with a person’s ability to understand reality and are associated with significant therapeutic and approach-related challenges [5], blunted affect, loss of motivation, the tendency to isolation [6], and several other negative symptoms pose an even more significant impact on the disease’s course, prognosis, and therapeutics [7]. Even though cognitive deficits are not listed as diagnostic symptoms for psychosis from the schizophrenia spectrum, they appear before the disease manifests clinically and seem to be directly connected with poor functionality [8]. Patients with schizophrenia present considerable overall impairment in cognition that is around two standard deviations below that of the general population [9].

Cognitive impairments affect different domains of cognition [10,11]. Among them, visual and verbal memory, executive function, working memory, cognitive flexibility, and reasoning are often related to diagnosis, disorganized thinking, and behavior dimensions and are frequently associated with poor prognosis [12,13]. Processing speed and social acuity are influenced by different psychopathological factors. Some research identifies processing speed as one of the most impaired cognitive domains in schizophrenia, making it a key predictor of overall cognitive performance. This is because processing speed directly impacts performance in various other areas, including symbol-coding, visual–perceptual ability, attention, and reaction time [14,15]. Regarding social cognition, people who have schizophrenia-related psychosis report a struggle to understand and respond appropriately to social cues that prevent them from engaging in and maintaining relationships, interacting in social groups, recognizing emotions, and communicating appropriately [16,17]. Eventually, literature data showed that cognitive deficits appear before illness onset [10] and seem relatively stable over time [7]. Although a general impairment was observed in psychosis, some of the domains appear more affected explicitly than the others, such as attention [18,19,20], memory [21,22], reasoning [20,23,24], executive function [19,21], and processing speed [18,25], which are mainly related to schizophrenia and affective psychosis.

As cognitive processes involve mainly the prefrontal cortex (PFC) and dopamine (DA) as monoamine mediator, the gene for catechol-O-methyltransferase (COMT) plays an essential role in modulating cognition [26,27,28]. COMT is a major enzyme linked to catecholamine degradation, catalyzing a methyl group’s transfer on the catechol nucleus from S-adenosyl-methionine to a hydroxyl group [29,30]. The gene encoding the enzyme is located on the 22q11 chromosome. Due to a single nucleotide polymorphism (SNP) rs4680 leading to a transition from guanine to adenine, resulting in a valine (Val) to methionine (Met) substitution at the codon 158, COMT enzymatic activity is changed. Val158Met occurs in around 50% of the general population of Europe and North America, while the homozygote variants Val/Val and Met/Met are only 20% and 30%, respectively [31]. Regarding enzymatic activity, the ValVal variant (GG genotype) has high enzymatic activity, the MetVal variant (AG genotype) presents intermediate activity, and the MetMet (AA genotype) has low enzymatic activity [32]. Consequently, prefrontal DA availability is influenced by the COMT genotype, with persons with AG and AA genotypes having intermediate to high dopamine release in PFC and better performance in cognitive tests. In addition, COMT rs4680 has been associated with psychotic-like experiences and executive dysfunction in previous studies [33]. The molecular genetics of cognition attributed a significant impact of COMT Val158Met effect on memory and executive function explained by the fact that the A allele increases tonic dopamine transmission with an impact on maintaining the relevant information; in contrast, the G allele increases phasic dopamine release with an impact on the flexibility of constantly updating new information [34]. Although literature data show better cognitive functions in Met carriers, Val homozygotes showed particularly better scores at decision-making tests related to lower dopamine levels based on decreased impulsivity [35]. On the contrary, the G allele was associated with reduced prefrontal activity, resulting in poor working memory, mainly attributed to stress methylation of the DNA [36].

The neuregulin 1 (NRG1) gene is located on chromosome 8p12, and its role as a susceptibility gene for schizophrenia [37] may be explained by the variety of processes of neuronal development and function that it modulates. N-methyl-D-aspartate (NMDA) glutamate and acetylcholine receptor modulation [38] may impact cognitive functions [39]. Moreover, NRG1 is involved in neuron migration, especially in gamma-aminobutyric-acid (GABA)-producing neurons, the most important central nervous system (CNS) inhibitor neurons. Several other essential functions are the myelinization of the neurons, brain development, and function that influence brain volume changes and may result in abnormalities in the circuits of the cerebellum, left hippocampus, and right anterior cingulate nucleus [39,40,41,42]. Murine models showed a significant impact of NRG1 in spatial cognition and social behavior [39], while other studies found an association with attention deficits [43] and visual memory deficits [44] in schizophrenia patients. Another candidate variant of susceptibility for schizophrenia is the rs3924999 variant of NRG1 [43,45]. Although literature data on its involvement in cognitive functions related to psychosis are limited, several studies found a potential involvement of rs3924999 genotypes in spatial memory and cognitive performance [46]; at the same time, its role in prepulse inhibition suggested that this variant could be an endophenotype for schizophrenia [47]. Prepulse suppression of the auditory startle reflex is a sensorimotor gating measure that is sometimes compromised in individuals with schizophrenia as a result of long-term over-activation of dopamine D2-like receptors [48].

The relationship between various categories of schizophrenia spectrum disorders and cognitive dysfunction is a subject of significant research interest. However, the influence of genetic polymorphisms, specifically COMT and NRG1, on this interaction remains poorly understood. This study hypothesizes that specific genetic polymorphisms in the NRG1 (rs3924999 and rs35753505) and COMT (rs4680) genes are associated with cognitive dysfunction in patients with schizophrenia spectrum disorders. More specifically, the study aims to determine whether these polymorphisms have a significant impact on cognitive performance, as measured by the Cognitive Neuropsychological/Neurocognitive Tests battery (CNS Vital Signs), in this population.

## 2. Material and Methods

### 2.1. Study Design

With a 5% margin of error and a 99% confidence level, the sample size was determined to match the 1% reported prevalence of schizophrenia in the general community [49]. This meant that at least 27 people were needed to achieve significance for the population study. A choice of 80% statistical power was made.

Patients with SSD, as defined by the DSM-5, participated in the cross-sectional research that we conducted. This study recruited participants from the Psychiatric Ward of Timisoara, Romania’s “Pius Brinzeu” County Emergency Hospital, between November 2021 and January 2024. Patients ranging in age from 19 to 67 gave their consent to take part in the trial. The current study’s inclusion criteria were as follows: (1) individuals who met the DSM-5 criteria for an acute episode of psychosis from the schizophrenia spectrum or who had been diagnosed with chronic psychosis for at least three years before the study, and (2) consenting to participate in the study and signing an informed consent form. The following served as representations of the exclusion criteria: (1) patients receiving treatment with any medication that might interfere with the assessment of the genotype’s influence on the cognitive evaluation; (2) individuals with co-occurring conditions (neurological illnesses, hepatic and renal disorders, cognitive evaluation); and (3) those with a history of drug use, diagnosed with drug-induced psychosis, or those who had used drugs one month before enrolling. Clinical and sociodemographic information was gathered from the recruited individuals.

The following made up the PICO model for the current investigation: P (population): individuals with SSD according to DSM-5 diagnostic criteria; I (issue): cognitive functions; C (comparison): individuals with AA > AG > GG for rs4680 (COMT); TT > TC > CC for rs35753505, and AA > AG > GG for NRG1 rs3924999 (NRG1); and O (outcomes): cognitive performance (as reported by CNS VS).

### 2.2. Ethics

The purpose of the study and its implications were explained to the patients prior to enrollment. They provided their written, informed consent to participate. The Helsinki Declaration Guidelines for scientific studies involving human participants were followed in conducting the investigation. With the number 10, the “Victor Babes” University of Medicine and Pharmacy in Timisoara granted ethical approval in 2021.

### 2.3. Clinical Assessment

Using PANSS, two independent researchers (M.B. and L.D.) assessed the psychotic symptoms of the patients in a blinded manner with respect to their genotype. Thirty components make up the evaluation scale, which is graded on a 7-degree scale from 1 (absent) to 7 (severe). The Positive Scale (7 items), Negative Scale (7 items), and General Psychopathology Scale (16 items) are the three subscales that make up the PANSS. There are 30 to 280 possible points in the total score. The Positive and Negative subscales have a score range of 7 to 49 points, while the General subscale has a score range of 16 to 112 points. PANSS’s internal consistency was deemed sufficient, with Cronbach’s alpha values ranging from 0.70 to 0.85.

In the current study, alongside cognitive testing and genotyping, we utilized the PANSS total score and subscale scores (PANSS T-PANSS total score; PANSS P-the score for the subscale P; PANSS N- the score for subscale N; PANSS G-the score for subscale G).

### 2.4. Cognitive Impairments Evaluation

The cognitive functions of the group were evaluated using the CNS Vital Signs. This is a non-invasive procedure that assesses a broad spectrum of brain function domains in an objective manner. The assessment was performed on time, with clear instruction presented before each part of the test. The domains of the tests included in the study were the following: verbal memory, visual memory, reaction time, processing speed, cognitive flexibility, reasoning, social acuity, and working memory. The neurocognitive index was calculated for each subject, representing a sum of all the domains included. We used the standard scores for each patient when performing the analysis. The scores for each domain can be separated into the following groups according to cognitive performance: values below 70 points were considered very low, between 70 and 79 points were considered low, between 80 and 90 points were representative of a low average performance, between 90 and 110 points were representative for an average cognitive performance, and scores higher than 110 points were considered above average. Meager results correspond to deficits and impairments, low scores correspond to moderate deficits and impairments, low average scores correspond to slight deficits and impairments, average scores correspond to normal function and capacity, and above average scores correspond to high functions and high capacity. CNS Vital Signs (CNS VS) has proven its good validity and reliability in numerous studies [50,51], which highlighted its essential role as a clinical screening instrument for cognitive impairments [52].

In order to account for potential cognitive differences related to age, all patients underwent a preliminary cognitive screening using the Mini-Mental State Examination (MMSE) prior to the more comprehensive cognitive testing with the CNS Vital Signs (CNS VS) battery. The MMSE ensured that participants met the cognitive criteria necessary for inclusion in the study. Additionally, the CNS VS battery evaluates cognitive performance using age-adjusted norms, allowing for standardized comparisons across individuals of different ages and ensuring that age-related differences did not confound the assessment of cognitive function.

### 2.5. NRG1 and COMT Genotyping

A 2 mL sample of peripheral blood was collected for genetic analysis. Genomic DNA extraction was performed following the instructions provided by the MagCore Nucleic Acid Extraction Kit (RBC Bioscience, New Taipei City, Taiwan). The DNA was preserved at −20 degrees Celsius. The concentration of DNA was measured using the Epoch Microplate Spectrophotometer (Agilent BioTek EPOCHSN, Santa Clara, CA, USA). For this study, one variant (rs4680) of the *COMT* gene and two variants (rs35753505 and rs3924999) of the NRG1 genes were selected for examination. TaqMan Drug Metabolism Genotyping Assays (Applied Biosystems Assay ID: C__25746809_50, ID: C____216209_50, ID: C____359159_10) and TaqMan Genotyping Master Mix (Applied Biosystems, Foster City, CA, USA) were utilized according to the manufacturer’s protocol. Purified DNA was amplified via real-time polymerase chain reaction (PCR) on the LightCycler 480 (Roche, Basel, Switzerland). Gene Scanning software version 1.5.1 (Roche) was employed. The genotyping process was conducted in a double-blind manner by two laboratory personnel. For quality control, 5% of the samples were selected randomly. The genotyping of ten samples was repeated with 100% reproducibility. Patients were categorized based on the genotype, as follows: for COMT rs4680, the genotypes were AA, AG, and GG; for NRG1 rs35753505, the genotypes were CC, CT, and TT; for NRG1 rs3924999, the genotypes were AA, AG, and GG.

### 2.6. Statistic Analysis

Descriptive and inferential statistics were conducted using the programming language R, version 4.3.2, with the R Studio 2023.12.0 environment, Posit Software, PBC “Ocean Storm” Release (17.12.2023) for Windows; Mozilla/5.0, Chrome/116.0.5845.190, Electron/26.2.4, and Safari/537.36. The mean and standard deviation were used to represent continuous variables, and the absolute and percentage values were used to represent categorical variables. The variables’ normality was evaluated using the Shapiro–Wilk test. The Kruskal–Wallis test was used for non-normally distributed data, and the ANOVA test was used for normally distributed variables to ascertain group differences. For non-normally distributed data, a post hoc Dunn’s test was performed to identify groups with significant differences, while the Tukey HSD test was used for normally distributed data to determine which groups pairs exhibited significant differences. The link between a qualitative dependent variable and two or more independent variables was estimated using multiple logistic regression. To find data points that significantly differed from the rest of the data, outlier tests were run. To evaluate the equality of variances for variables derived for two or more groups, the Levene test was used. With a significance level of α = 0.05, the statistical power was fixed at 80%.

## 3. Results

### 3.1. Descriptive Analysis

**Figure 1:**
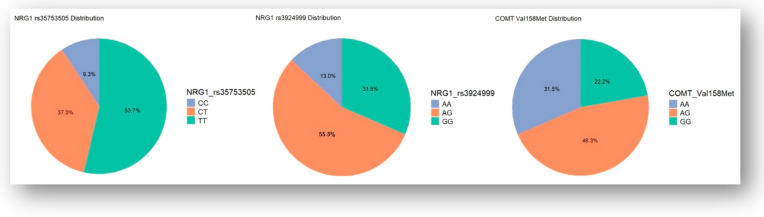
Genetic polymorphisms of the study lot.

A total of fifty-four subjects were included in the study. Genetic polymorphisms of rs35753505 (CC; CT; TT), rs4680 (AA; AG; GG), and rs3924999 (AA; AG; GG), having been evaluated, are illustrated in Figure 1.

Participants were divided into four categories based on their diagnosis: those experiencing their first episode of psychosis with a diagnosis of schizophreniform disorder, as well as patients with schizophrenia, schizoaffective disorder, and delusional disorder. Sociodemographic data are represented in Table 1. The groups’ mean ages differed significantly, according to statistical analysis (*p* = 0.001). At a mean age of 32 years old (SD = 11), patients with schizophreniform illness accounted for 44.44% of the sample. The mean age of the patients with schizophrenia was 37 years old (SD = 8), accounting for 20.37% of the total participants. The mean age of the subjects diagnosed with schizoaffective disorder was 39 years old (SD = 8), accounting for 22.22% of the sample; in contrast, the mean age of the patients diagnosed with delusional illness was 55 years old (SD = 10), representing only 12.96% of the sample.

Table 2 exemplifies the clinical characteristics of the population. For patients with schizophreniform disorder, the mean age of onset was 31 years (SD = 12); for patients in the schizophrenia group, it was 27 years (SD = 6); for subjects with schizoaffective disorders, it was 26 years (SD = 7); and for patients with delusional disorder, it was 37 years (SD = 7) (*p* = 0.03).

Considering the genetic polymorphisms of COMT rs4680 in the study group, in schizophreniform disorder patients, AG genotype was found in 46% of patients, followed by GG genotype (29%) and AA genotype (25%). The percentages for schizophrenia, schizoaffective, and delusional disorder were similar, with no statistically significant differences (*p* = 0.7). Regarding NRG1 rs35753505, the most represented genotype was TT, followed by CT and CC, with no significant differences between the diagnostic categories (*p* = 0.56). Lastly, NRG1 rs3924999 was dominantly represented by AG genotypes, followed by GG and AA genotypes, with no statistically significant differences between the groups according to diagnostic (*p* = 0.19).

Following statistical analysis of the mean scores for cognitive functions according to diagnostic categories, significant differences were found with respect to the neurocognitive index, reaction time, processing speed, and executive function after Bonferroni correction and the results are illustrated in Table 3.

### 3.2. Genetic Polymorphisms of COMT rs4680 and NRG 1 (rs3924999 and rs35753505) on Cognitive Domains in SSD: General and Particular Aspects

Following the performed statistical analysis, we investigated the impact of COMT rs4680 and NRG1 (rs3924999 and rs35753505) polymorphisms on cognitive performance in schizophrenia spectrum disorders (SSDs). The analysis was conducted both across the entire sample, irrespective of diagnosis, and within specific diagnostic categories to capture both general and particular genetic influences. In the general analysis, we explored the relationship between these genetic variants and key cognitive domains, identifying patterns of cognitive impairment that were consistently associated with the studied polymorphisms. Subsequently, we examined how these genetic effects varied according to diagnosis and illness stage (acute vs. chronic), revealing diagnosis-specific influences on cognitive performance. This approach allows for a comprehensive understanding of the genetic contributions to cognitive dysfunction in SSD, highlighting both universal patterns and more distinct genetic effects in different patient subgroups.

The statistical analysis conducted included all cognitive domains represented in the descriptive statistics to provide a comprehensive overview of cognitive performance across the sample. However, many of the results did not reach statistical significance, likely due to the limited statistical power in some analyses. To ensure clarity and relevance, we have chosen to present only the statistically significant findings and those that showed meaningful trends in the detailed statistical results. This approach focuses on the most relevant and interpretable outcomes, while ensuring that the key cognitive domains impacted by the genetic polymorphisms are highlighted.

#### 3.2.1. Relation Between Genotypes and Cognitive Performance across SSD Spectrum

**Figure 2:**
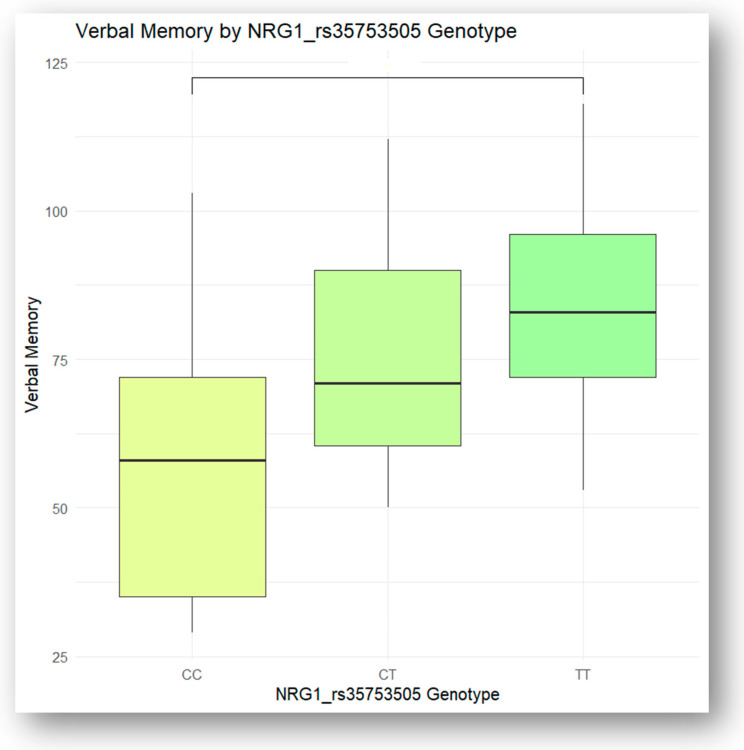
Verbal memory of the NRG1 rs35753505 genotypes.

Following the ANOVA test analysis, significant differences in verbal memory were found between the NRG1 rs35753505 genotypes, with Tukey’s HSD post hoc test identifying the specific group pairings (TT, CC, and CT) that exhibited significant differences, as illustrated in Figure 2. The scores on verbal memory were lower for CC genotypes when compared to CT genotypes and TT genotypes, but with no statistical significance (*p*-adj = 0.19 and *p*-adj = 0.46, respectively). Additionally, the analysis revealed significant differences between the TT and CC genotypes, with TT > CC (*p*-adj = 0.03).

A logistic regression analysis, illustrated in Table 4, was conducted to examine the association between NRG1 rs35753505 genotypes (CC, CT, TT) and verbal memory performance. The results indicated that the TT genotype was significantly associated with better verbal memory performance (*p*-adj = 0.033), while the CC and CT genotypes showed negative associations that were not statistically significant (*p*-adj = 0.094 and *p*-adj = 0.326, respectively). These findings suggest that individuals with the TT genotype may have a cognitive advantage in verbal memory compared to those with the CC or CT genotypes.

The logistic regression analysis assessing the relationship between NRG1 rs35753505 genotypes and social acuity performance is detailed in Table 5. The results indicated that the TT genotype was significantly associated with lower social acuity (*p*-adj = 0.04), suggesting a potential negative impact of this genotype on social skills. In contrast, the CC and CT genotypes showed no statistically significant associations with social acuity (*p*-adj = 0.601 and *p*-adj = 0.124, respectively), indicating no strong evidence of their influence on social performance.

A logistic regression analysis was performed to examine the association between NRG1 rs3294999 genotypes and reaction time performance. The analysis revealed that the GG genotype was significantly associated with faster reaction times (*p*-adj = 0.035), indicating a potential positive impact on this cognitive function. The AG genotype showed a non-significant trend towards slower reaction times (*p*-adj = 0.098), while the AA genotype displayed a minimal and non-significant effect on reaction time (*p*-adj = 0.464). These findings suggest that the GG genotype may confer an advantage in reaction speed compared to the AA and AG genotypes. The results are represented in Table 6.

**Figure 3:**
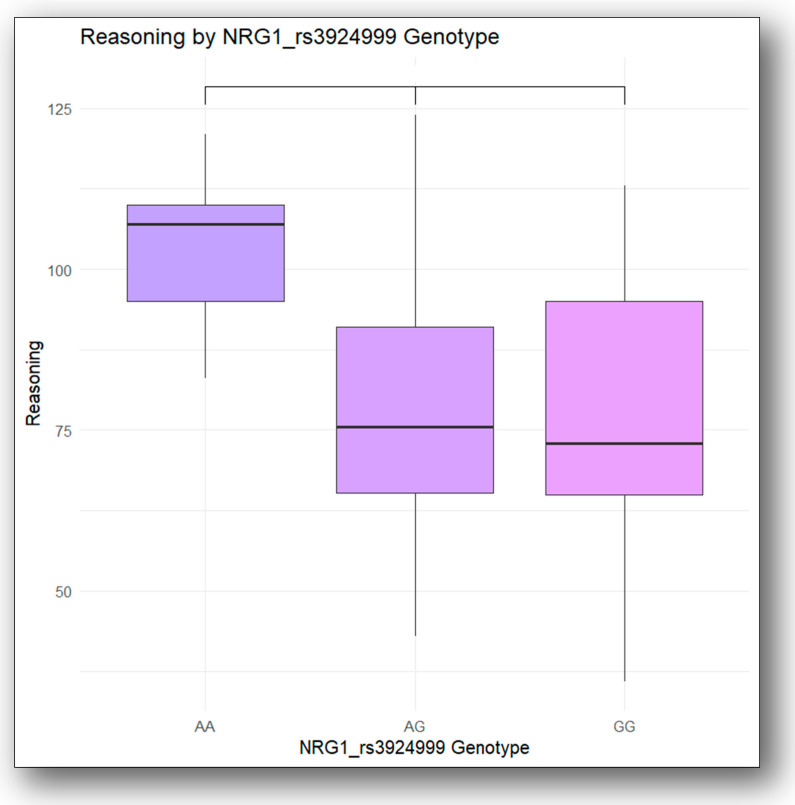
The differences in the reasoning of the NRG1 rs3924999 genotypes.

Significant differences were found between the AG and AA genotypes and between the GG and AA genotypes when ANOVA analysis was performed, with Tukey’s HSD post hoc test identifying the specific group pairings, where the AA genotypes had higher mean reasoning scores than the AG genotypes (*p*-adj = 0.01) and the GG genotypes (*p*-adj = 0.02) (AA > GG; AA > AG). On the other hand, GG and AG did not differ significantly, despite the fact that AG patients scored higher on reasoning than GG patients (*p*-adj = 0.98). These results are presented in Figure 3.

A logistic regression analysis was conducted to explore the relationship between NRG1 rs3294999 genotypes and reasoning performance and the results are presented in Table 7. The analysis showed that the AA genotype was significantly associated with better reasoning performance (*p*-adj = 0.01), suggesting a positive impact of this genotype. The AG genotype exhibited a non-significant trend towards poorer reasoning (*p*-adj = 0.09), while the GG genotype showed no significant effect on reasoning (*p*-adj = 0.892). These findings indicate that the AA genotype may enhance reasoning ability compared to the AG and GG genotypes. These results complement the ANOVA analysis.

The statistical analysis conducted on visual memory, cognitive flexibility, processing speed, executive function, and working memory in relation to the genetic polymorphisms of COMT and NRG1 did not yield statistically significant results. As these findings did not meet the threshold for significance, they are not included in the detailed results section. Given the lack of notable trends or relevance, the data for these cognitive domains are not shown in the text or tables. Where applicable, relevant statistical tests were performed, but no significant associations were observed between the polymorphisms and the mentioned cognitive domains.

#### 3.2.2. The Role of Genetic Polymorphisms on Cognitive Functions in Relation to the Illness Stage (Acute vs. Chronic)

The interaction ANOVA test on verbal memory performance related to NRG1 rs35753505 genotypes, grouped by diagnosis, yielded significant results. Tukey’s HSD post hoc analysis revealed that only patients with the TT genotype had significantly higher mean scores compared to those with the CC genotype (*p*-adj = 0.03), regardless of diagnosis. No significant differences were found between the TT and CT genotypes after Tukey’s HSD.

When analyzing reasoning in relation to NRG1 rs3924999 genotypes, an interaction ANOVA was performed as the statistical analysis, revealing that subjects with AG and GG genotypes had significantly lower mean scores compared to those with the AA genotype (*p*-adj = 0.01 and *p*-adj = 0.02, respectively), regardless of diagnosis. Tukey’s HSD post hoc test was conducted for each genotype comparison. Additionally, the statistical analysis comparing chronic patients with schizophrenia and schizoaffective disorder to those with schizophreniform disorder (first episode of psychosis) also involved an interaction ANOVA, which revealed several significant differences in cognitive test performance based on genotype, with Tukey’s HSD used to identify specific group differences.

**Figure 4:**
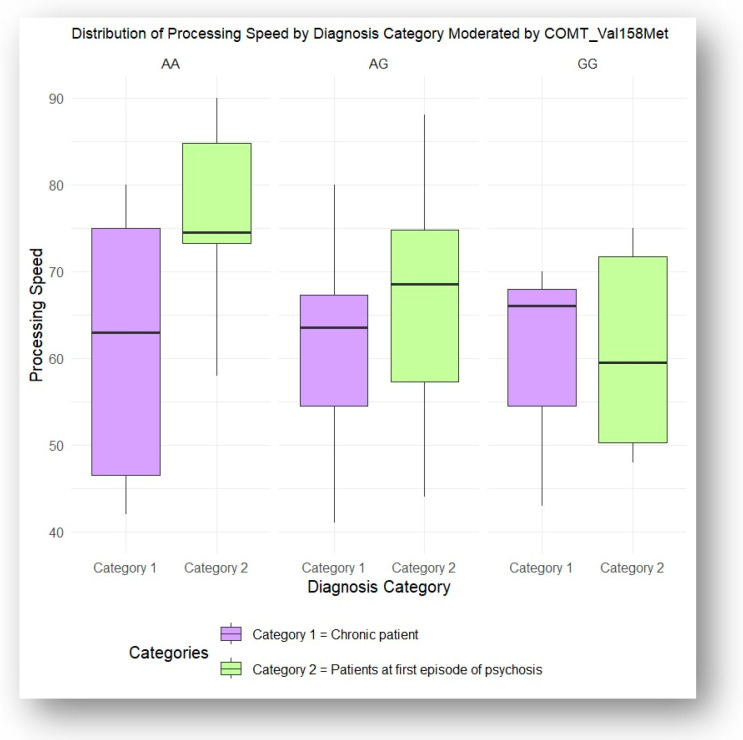
Processing speed influenced by COMT rs4680 genotypes, according to the illness stage (acute vs. chronic).

An interaction ANOVA revealed that the COMT Val158Met polymorphism significantly impacted patients’ processing speed scores, as detailed in Figure 4. The analysis showed that patients with schizophreniform disorder had significantly higher mean scores compared to those with schizophrenia and schizoaffective disorder (*p* = 0.01). Tukey’s HSD post hoc test was performed to identify the specific group differences between the diagnostic categories.

**Figure 5:**
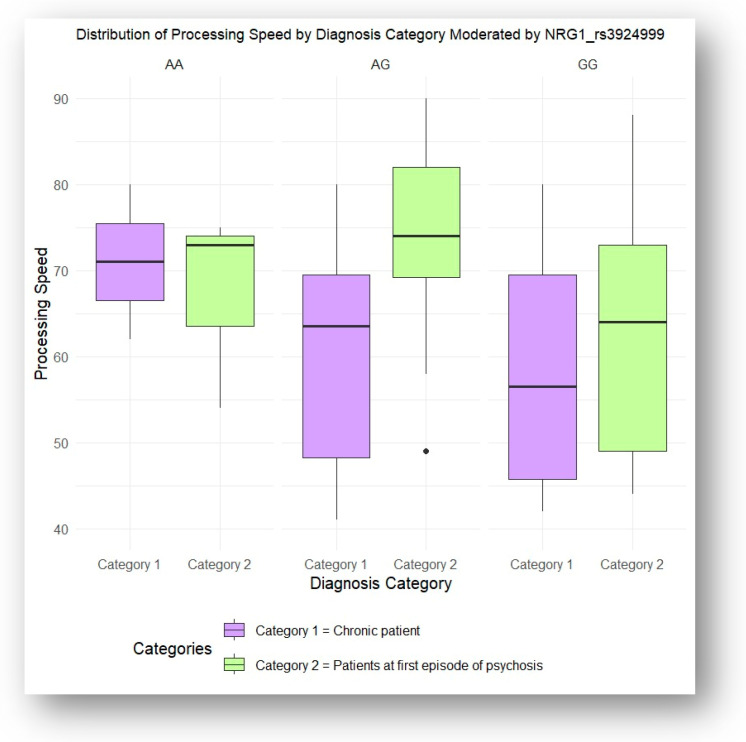
Processing speed influenced by NRG1 rs3924999 genotypes, according to the illness stage (acute vs. chronic).

**Figure 6:**
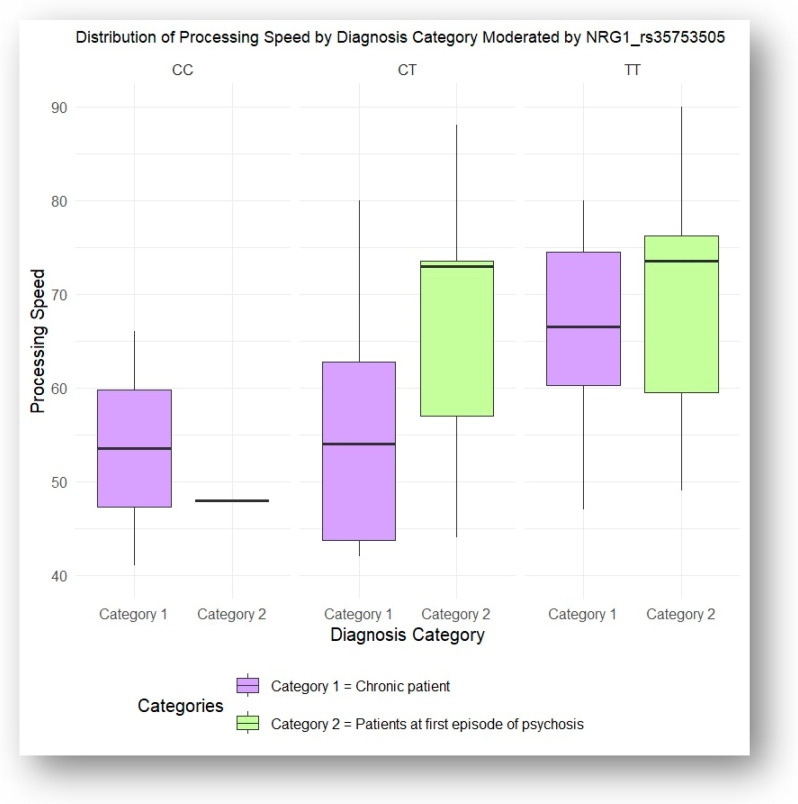
Processing speed influenced by NRG1 rs35753505 genotypes, according to the illness stage (acute vs. chronic).

Similarly, interaction ANOVA analysis demonstrated that the NRG1 rs3924999 and NRG1 rs35753505 genotypes significantly influenced performance in the processing speed domain based on diagnostic category. Patients experiencing their first episode of psychosis showed significantly better processing speed performance compared to chronic patients (*p* = 0.006 and *p* = 0.02, respectively). Tukey’s HSD post hoc tests were conducted to identify the specific differences between these groups, as illustrated in Figure 5 and Figure 6.

An interaction ANOVA revealed that NRG1 rs3924999 significantly impacted cognitive flexibility scores, with chronic patients showing significantly lower scores compared to those experiencing their first episode of psychosis (*p*-adj = 0.03). Tukey’s HSD post hoc test was performed to identify the specific differences between these groups.

#### 3.2.3. The Role of Genetic Polymorphisms on Cognitive Functions in Relation to the Diagnosis Category

For specific diagnostic categories such as schizophrenia, schizoaffective disorder, schizophreniform disorder, and delusional disorder, the analysis of genetic polymorphisms in relation to cognitive functions did not reach statistical significance. This is likely due to the smaller sample sizes when patients were grouped by these individual diagnoses, which could affect the generalization of the data. As a result, the data for these specific diagnostic categories are not presented in the detailed results, given the lack of significant findings. These limitations highlight the need for larger sample sizes to adequately explore the potential genotype–cognition relationships within each diagnostic subgroup.

## 4. Discussion

This study investigated the association between NRG1 (rs3924999 and rs35753505) and COMT gene (rs4680) polymorphisms and various cognitive functions in patients with schizophrenia spectrum disorders, using the CNS Vital Signs battery. The findings provide valuable insights into how specific genotypes may influence cognitive performance in these patients, with potential implications for clinical practice.

The significant impact of the COMT Val158Met polymorphism on processing speed, particularly in patients with schizophreniform disorder, offers a compelling insight into the cognitive differences between early and advanced stages of psychotic disorders. Patients with schizophreniform disorder, who are experiencing their first episode of psychosis, demonstrated higher mean processing speed scores compared to those with chronic schizophrenia and schizoaffective disorder. This suggests that cognitive functions, particularly processing speed, may be better preserved in the early stages of illness, which is critical for everyday cognitive tasks such as decision-making and problem-solving. Processing speed has been identified in the literature as one of the most impaired cognitive domains in schizophrenia and is a strong predictor of overall cognitive functioning, influencing tasks such as symbol coding, visual-perceptual ability, attention, and reaction time [14,15]. The link between *COMT* gene variation and cognitive performance at different stages of the illness suggests that genetic factors may contribute to cognitive decline as the disease progresses. These findings highlight the potential clinical value of early interventions aimed at preserving cognitive function, particularly processing speed, in patients at risk of developing chronic schizophrenia or schizoaffective disorder. Early, personalized treatment strategies targeting cognitive decline could improve functional outcomes and delay further cognitive deterioration [53], making this an essential consideration for managing early-stage psychosis.

Among cognitive domains affected by schizophrenia, working memory is one of the most specific to schizophrenia. In a study by Anand et al., patients with CC genotypes performed poorly on verbal fluency, visual memory, and working memory, yet scored higher in executive function [54]. Although we could not find evidence for working memory performance, the logistic regression analysis revealed that the TT genotype of NRG1 rs35753505 is significantly associated with better verbal memory performance, as compared to the CC genotype. Despite the fact that NRG1 rs35753505 is the most reported single marker for schizophrenia [55,56], a clear role of its genotype related to cognitive function in this disorder has not been established yet. Another study investigating cognitive functions in patients suffering from schizophrenia and the impact of NRG1 rs35753505 on cognition found similar correlations between genotype status and semantic verbal fluency. Specifically, patients homozygous for the C-allele (CC) performed worse on verbal fluency tasks compared to those carrying the T-allele (TT or CT genotypes) [57]. Similarly, Almeida et al. reported that subjects with the CC genotypes exhibited poorer performance in both verbal memory and visual episodic memory compared to those with CT and TT genotypes. These findings are consistent with our results regarding verbal memory but differ from our observations on visual memory [58]. This suggests that individuals with the TT genotype may have a cognitive advantage in verbal memory, which could have implications for cognitive rehabilitation strategies in schizophrenia. Interestingly, the existing literature suggests that the NRG1 rs35753505 genotype modulates brain activation during episodic memory tasks, with CC genotype carriers exhibiting hyperactivation in several brain areas involved in encoding and retrieval processes [43,59]. Another important mechanism explaining the complex role of neuregulin 1 in cognitive functions of schizophrenia spectrum patients relies on its risk C-alleles, which were associated with the expression of NMDA receptors with a negative impact on cognition [60]. It is important to interpret these findings cautiously, as the cited study involved subjects without psychiatric diagnoses, who may differ genetically from patients with SSDs. However, the literature comparing the cognitive outcomes of TT genotype carriers versus other genotypes in similar populations should be further explored to validate these findings.

In contrast to the positive effects of the TT genotype on verbal memory, our analysis indicated that this genotype is associated with lower social acuity. This finding highlights the complex and sometimes contradictory effects of genetic variations on different cognitive domains. The clinical significance of this result is noteworthy, as social acuity is critical for social functioning in schizophrenia patients [61]. Further research should examine whether this genotype could serve as a biomarker for predicting social deficits and whether targeted interventions could mitigate these effects.

Considering NRG1 rs3294999, the GG genotype was significantly associated with faster reaction times, suggesting a potential cognitive benefit in terms of processing speed in diverse contexts. This finding aligns with the idea that certain NRG1 variants may enhance specific cognitive abilities. Given that reaction time is crucial for daily functioning and social interactions, these results could inform strategies to improve cognitive outcomes in patients with schizophrenia, particularly those carrying the GG genotype. Future studies should compare these findings with other populations to assess the generalizability of these results.

Further, both the logistic regression and ANOVA analyses consistently indicated that the AA genotype of NRG1 rs3294999 is associated with better reasoning performance compared to the AG and GG genotypes. This suggests that the AA genotype may confer a protective effect on reasoning abilities, which are often impaired in schizophrenia. This finding is particularly important for clinical practice, as reasoning skills are linked to functional outcomes in schizophrenia [62]. Further literature review is necessary to compare these findings with other studies and to explore the potential mechanisms underlying this association.

Although NRG1 rs3294999 is considered a risk gene for schizophrenia, the present study found few statistically significant differences in reasoning and cognitive flexibility scores between patients with onset psychosis and chronic patients when comparing its genotype. Thus, chronic patients scored lower than patients at the first episode. Moreover, subjects with AA genotypes scored better in reasoning than those with AG and GG genotypes, regardless of diagnostic category. Other studies suggest that NRG1 rs3294999 could affect attention and reasoning ability after risperidone treatment, showing that GG genotypes tended to have lower scores in attention and reasoning when compared to AA and AG genotypes [45,47].

Thus, the identification of specific genetic polymorphisms, particularly NRG1 rs35753505, NRG1 rs3294999, and COMT rs4680 may help influence individual responses to treatment. This knowledge can guide clinicians in tailoring antipsychotic treatments based on a patient’s genetic profile, potentially improving treatment outcomes. Now, multigene panels that analyze multiple genes or variants in a single assay are widely available and offered by many clinical laboratories. These panels are more cost-effective and provide valuable preemptive pharmacogenetic information applicable to various medications a patient might receive over their lifetime [63]. Additionally, cognitive rehabilitation has been shown to effectively reduce cognitive impairment, thereby improving the quality of life in patients [64,65].

Hence, understanding the impact of genetic polymorphisms on cognitive performance allows for the development of targeted cognitive rehabilitation strategies [65,66]. For example, patients with NRG1 rs35753505 TT genotypes may benefit from specific cognitive interventions aimed at enhancing cognitive flexibility and visual memory, while NRG1 rs35753505 CC or CT genotypes may benefit from interventions aiming social acuity. Strategies targeting the COMT Val158Met polymorphism could focus on improving processing speed, potentially enhancing prognosis and functionality. Similarly, interventions can be designed to address the specific cognitive deficits associated with different NRG1 rs3294999 genotypes. The findings suggest that different genotypes may impact treatment response differently, implying the necessity for genotype-specific therapeutic approaches.

Interestingly, literature data revealed that after transcranial direct current stimulation, a positive effect was observed on auditory hallucination in patients with GG genotypes of COMT rs4680 and AA genotypes of NRG1 rs35753505 [67]. Not only do rs35753505 genotypes show a strong impact on neuroplasticity in schizophrenia, but they also seem to substantially impact cognitive functions in general in patients who do not receive antipsychotic treatment [68] and reasoning and attention after risperidone treatment [69].

Likewise important, these genetic variations appear to significantly affect neuroplasticity, cognitive functions, and treatment outcomes, such as improvements in auditory hallucinations and reasoning skills. Since schizophrenia is a highly heterogeneous disorder with diverse cognitive and symptom profiles, identifying patients’ genotypes allows clinicians to personalize treatment plans. Tailoring therapies based on genetic profiles can optimize treatment efficacy [65], enhance cognitive performance, and reduce side effects by ensuring that interventions, whether pharmacological or non-pharmacological, align with the individual’s genetic predisposition. As a result, genotyping could be a valuable tool for guiding treatment strategies [70] and improving long-term outcomes in patients with schizophrenia.

Nonetheless, this study has several limitations. First, due to its cross-sectional design, it cannot control for potential cohort effects. Further, longitudinal studies are needed to observe changes in cognitive function over time in relation to treatment and genetic polymorphisms. Second, the relatively small sample size reduces the reproducibility of the results. Therefore, future studies should include additional variables to achieve higher predictive accuracy.

We intend to expand our investigation to a bigger group as a case–control study, including at least two cognitive evaluation sessions for each subject, since a larger sample size boosts statistical power. Future research directions could include exploring cognitive improvements in risperidone or clozapine-treated patients with SSD by incorporating a control group receiving no medication or an alternative atypical antipsychotic.

## 5. Conclusions

In conclusion, this study provides valuable insights into the impact of NRG1 (rs35753505 and rs3924999) and COMT (rs4680) polymorphisms on cognitive performance in schizophrenia spectrum disorders. The notable influence of the COMT Val158Met polymorphism on processing speed, especially in schizophreniform disorder patients, underscores its importance as a key factor for early-stage clinical interventions. Additionally, the TT genotype of NRG1 rs35753505 was significantly associated with better verbal memory but lower social acuity, highlighting the complex role of this gene in different cognitive domains. The NRG1 rs3294999 GG genotype was linked to faster social reaction times, while the AA genotype showed better reasoning abilities, suggesting cognitive advantages based on genotype. These findings underscore the potential for personalized cognitive interventions and treatment strategies in schizophrenia, emphasizing the clinical relevance of genetic profiling. However, larger, longitudinal studies are needed to confirm these associations and explore their long-term effects on treatment outcomes.

## Figures and Tables

**Table 1:** Sociodemographic data of the study.

Variables ^†^	Schizophreniform Disorder (n = 24)	Schizophrenia (n = 11)	Schizoaffective Disorder (n = 12)	Delusional Disorder (n = 7)	*p*-Value
**Age** (mean ± SD)	32 ± 11	37 ± 8	39 ± 8	55 ± 10	0.001 *
**Sex**					0.01
Male	67% (16)	82% (9)	25% (3)	29% (2)	
Female	33% (8)	18% (2)	75% (9)	71% (5)	
**Education (years of study)** (mean ± SD)	12 ± 1	13 ± 2	13 ± 1	13 ± 3	0.4
**Occupation**					0.01
Student	17% (4)	0% (0)	0% (0)	0% (0)	
Employed	37% (9)	36% (4)	33% (4)	57% (4)	
Unemployed	42% (10)	36% (4)	17% (2)	0% (0)	
Retired	0% (0)	0% (0)	0% (0)	14% (1)	
Ill-health retired	4% (1)	28% (3)	50% (6)	29% (2)	

SD—standard deviation; *p* *—the value of *p* was statistically significant after Bonferroni correction; α value after Bonferroni correction = 0.012; ^†^ age and education are represented as means and standard deviation; gender and occupation are shown as percentages with absolute values expressed in brackets.

**Table 2:** Clinical characteristics of the lot.

Variables ^†^	Schizophreniform Disorder (n = 24)	Schizophrenia (n = 11)	Schizoaffective Disorder (n = 12)	Delusional Disorder (n = 7)	*p*-Value
**Age of onset** (mean ± SD)	31 ± 12	27 ± 6	26 ± 7	37 ± 7	0.03
**Number of episodes** (mean ± SD)	1 ± 0	5 ± 4	9 ± 6	5 ± 3	0.001 *
**PANSS** (mean ± SD)					
P	9 ± 6	9 ± 4	6 ± 6	7 ± 3	0.13
N	5 ± 4	4 ± 2	5 ± 4	4 ± 2	0.52
G	14 ± 8	10 ± 5	16 ± 9	7 ± 5	0.51
T	26 ± 14	22 ± 8	28 ± 13	18 ± 6	0.49
**COMT (rs4680)**					0.7
AA	25% (6)	27% (3)	50% (6)	29% (2)	
AG	46% (11)	45% (5)	42% (5)	57% (4)	
GG	29% (7)	27% (3)	8% (1)	14% (2)	
**NRG1 (rs3924999)**					0.19
AA	13% (3)	18% (2)	0% (0)	29% (2)	
AG	42% (10)	55% (6)	83% (10)	57% (4)	
GG	46% (11)	27% (3)	17% (2)	14% (1)	
**NRG1 (rs35753505)**					0.56
CC	4% (1)	18% (2)	17% (2)	0% (0)	
CT	42% (10)	18% (2)	42% (5)	43% (3)	
TT	54% (13)	64% (7)	42% (5)	57% (4)	

Psychotic symptoms measured by PANSS and subscales scores; SD—standard deviation; α value after Bonferroni correction = 0.006; *p* *—the value of p was statistically significant after Bonferroni correction; ^†^ age of onset, number of episodes, and PANSS scores are represented as means and standard deviation; genetic polymorphisms of COMT and NRG 1 are shown as percentages with absolute values expressed in brackets.

**Table 3:** Cognitive domains of the population.

CNS Vital Signs ^†^	Schizophreniform Disorder (n = 24)	Schizophrenia (n = 11)	Schizoaffective Disorder (n = 12)	Delusional Disorder (n = 7)	*p*-Value
**Neurocognitive index** (mean ± SD)	67 ± 21	71 ± 25	38 ± 23	59 ± 24	0.001 *
**Verbal memory** (mean ± SD)	77 ± 20	82 ± 17	72 ± 24	89 ± 17	0.27
**Visual memory** (mean ± SD)	84 ± 15	93 ± 19	75 ± 19	88 ± 20	0.09
**Reaction time** (mean ± SD)	55 ± 22	73 ± 24	35 ± 25	63 ± 25	0.001 *
**Cognitive flexibility** (mean ± SD)	61 ± 33	61 ± 41	21 ± 29	52 ± 23	0.02
**Processing speed** (mean ± SD)	72 ± 16	71 ± 12	48 ± 17	67 ± 16	0.001 *
**Executive function** (mean ± SD)	63 ± 30	63 ± 40	25 ± 27	57 ± 23	0.001 *
**Social acuity** (mean ± SD)	76 ± 24	59 ± 36	58 ± 45	64 ± 42	0.61
**Reasoning** (mean ± SD)	82 ± 21	96 ± 22	72 ± 18	77 ± 16	0.04
**Working memory** (mean ± SD)	85 ± 16	91 ± 12	79 ± 11	77 ± 12	0.07

SD—standard deviation; α value after Bonferroni correction = 0.005, *p* *—the value of p was statistically significant after Bonferroni correction; ^†^ values of cognitive scores for each domain are expressed as means and standard deviation.

**Table 4:** Logistic regression of NRG1 rs35753505 genotypes and verbal memory performance.

* Coefficient	Estimate	SE	Z-Value	*p*-adj-Value	2.5% CI	97.5% CI
CC	−0.088	0.052	−1.673	0.094	−0.22	−0.01
CT	−0.022	0.023	−0.982	0.326	−0.07	0.02
TT	−0.056	0.026	2.121	0.033	0.01	0.11

* McFadden’s R^2^ = 0.3; Coefficient (Estimate): Represents the effect size of the genotype on the outcome variable; SE (Standard Error): Indicates the variability in the estimate; Z-value: The test statistic measuring how far the coefficient is from zero; *p*-adj-value: Indicates the statistical significance of the effect; 2.5% CI/97.5% CI (Confidence Interval): The range within which the true effect is expected to fall with 95% confidence.

**Table 5:** Logistic regression of NRG1 rs35753505 genotypes and social acuity performance.

* Coefficient	Estimate	SE	Z-Value	*p*-adj-Value	2.5% CI	97.5% CI
CC	−0.013	0.026	0.522	0.601	−0.2	0.07
CT	−0.026	0.01	1.538	0.124	−0.01	0.06
TT	−0.03	0.01	−2.049	0.04	−0.07	−0.01

* McFadden’s R^2^ = 0.23; Coefficient (Estimate): Represents the effect size of the genotype on the outcome variable; SE (Standard Error): Indicates the variability in the estimate; Z-value: The test statistic measuring how far the coefficient is from zero; *p*-adj-value: Indicates the statistical significance of the effect; 2.5% CI/97.5% CI (Confidence Interval): The range within which the true effect is expected to fall with 95% confidence.

**Table 6:** Logistic regression of NRG1 rs3294999 genotypes and reaction time performance.

* Coefficient	Estimate	SE	Z-Value	*p*-adj-Value	2.5% CI	97.5% CI
AA	0.038	0.05	0.732	0.464	−0.01	0.01
AG	−0.03	0.02	−1.655	0.098	−0.07	0.01
GG	0.04	0.02	2.106	0.035	0.01	0.09

* McFadden’s R^2^ = 0.48; Coefficient (Estimate): Represents the effect size of the genotype on the outcome variable; SE (Standard Error): Indicates the variability in the estimate; Z-value: The test statistic measuring how far the coefficient is from zero; *p*-adj-value: Indicates the statistical significance of the effect; 2.5% CI/97.5% CI (Confidence Interval): The range within which the true effect is expected to fall with 95% confidence.

**Table 7:** Logistic regression of NRG1 rs3294999 genotypes and reasoning performance.

* Coefficient	Estimate	SE	Z-Value	*p*-adj-Value	2.5% CI	97.5% CI
AA	0.011	0.04	2.444	0.01	0.01	0.22
AG	−0.03	0.01	−1.648	0.09	−0.06	0.01
GG	0.01	0.01	−0.135	0.892	−0.03	0.03

* R^2^ = 0.35; Coefficient (Estimate): Represents the effect size of the genotype on the outcome variable; SE (Standard Error): Indicates the variability in the estimate; Z-value: The test statistic measuring how far the coefficient is from zero; *p*-adj-value: Indicates the statistical significance of the effect; 2.5% CI/97.5% CI (Confidence Interval): The range within which the true effect is expected to fall with 95% confidence.

## Data Availability

Data available on request.
